# Extemporaneous Formulations: Filling the Gap in the Pharmaceutical Industry with Personalized Medicines

**DOI:** 10.3390/pharmaceutics18020209

**Published:** 2026-02-06

**Authors:** Antonio Lopalco, Nunzio Denora

**Affiliations:** Department of Pharmacy—Pharmaceutical Sciences, University of Bari Aldo Moro, 4, E. Orabona Street, 70126 Bari, Italy

## 1. Introduction

The progressive shift of healthcare systems toward personalised and patient-centred therapeutic strategies has exposed the structural limitations of standardised pharmaceutical manufacturing. Although industrial drug development ensures high levels of quality, safety, and scalability, authorised medicinal products often fail to address the full spectrum of clinical needs encountered in practice. These gaps may result from the lack of appropriate dose strengths, rigid dosage forms, unsuitable excipient profiles, or the need for alternative routes of administration. In this context, extemporaneous pharmaceutical formulations remain an essential component of modern pharmacy practice, enabling the preparation of customised medicines tailored to individual patient requirements [1].

Across regulatory jurisdictions, pharmaceutical compounding is recognised as a legitimate professional activity performed by authorised healthcare professionals, primarily pharmacists, under a prescription or an established professional relationship. While legal contexts differ internationally, common principles are reflected in major pharmacopeial standards, including the European Pharmacopoeia and the United States Pharmacopeia–National Formulary (USP–NF), which define technical requirements and quality attributes for substances, excipients, and compounded preparations. Regulatory bodies such as the European Medicines Agency (EMA) and national competent authorities acknowledge compounding as necessary when industrially manufactured medicines are unavailable or unsuitable, provided it is conducted in accordance with professional standards and risk-based quality systems [2].

Extemporaneous formulations are relevant across all stages of life and in diverse clinical settings, including rare diseases, oncology, cardiology, dermatology, palliative care, and hospital therapies [3]. In this sense, compounding represents a practical implementation of personalised medicine at the point of care, allowing pharmacists and pharmaceutical scientists to translate formulation science into patient-specific therapeutic solutions [4].

Formulation development in extemporaneous compounding differs fundamentally from industrial pharmaceutical development. Compounded preparations are produced on demand, often within limited timeframes, and are not subject to centralised marketing authorisation. Consequently, their quality, safety, and suitability rely on professional competence, compliance with pharmacopeial standards, and adherence to beyond-use dating principles rather than pre-market regulatory approval [5]. Both the European Pharmacopoeia and USP–NF stress the control of critical quality attributes, including identity, purity, strength, stability, and microbiological quality, placing significant responsibility on the compounding pharmacist.

Within this context, pharmaceutical compounding strengthens the patient–physician–pharmacist triad and reinforces the integration of pharmaceutical sciences into clinical decision-making. Excipients play a crucial functional role by supporting solubilisation, stabilisation, preservation, and optimisation of drug delivery [6]. Their selection must be scientifically justified, pharmacopeia-compliant, and aligned with patient-specific needs to ensure safety and effectiveness.

This Special Issue, “Extemporaneous Formulations: Filling the Gap in the Pharmaceutical Industry with Personalized Medicines”, provides a multidisciplinary overview of the scientific, technological, clinical, and regulatory aspects of pharmaceutical compounding. Contributions address formulation development, dosage forms and excipients, stability, compounding technologies, quality control, and legislative frameworks across healthcare systems. Particular attention is given to the interface between pharmacy practice and emerging drug delivery technologies, highlighting innovation within actual compounding environments.

## 2. Articles in This Special Issue

This Special Issue includes eleven contributions that collectively demonstrate how extemporaneous formulations can fill therapeutic gaps when authorised medicines are unavailable, unsuitable, or require individual adaptation. Across diverse clinical applications and compounding practices, the papers converge on a shared principle: modern pharmaceutical compounding increasingly relies on structured quality systems, analytical evidence, and patient-centric design to ensure reproducible preparation and safe use.

A broad and highly informative overview of pharmacy preparations within a national healthcare context is provided by Woerdenbag and co-workers, who describe the organisation, scope, and product care responsibilities associated with pharmacy preparations in the Netherlands. By highlighting specialist areas such as paediatric dosage forms, dysphagia-related administration, reconstitution of biologicals, and radiopharmaceutical practices, the article offers a valuable reference model illustrating how compounding can be integrated into healthcare pathways while maintaining a strong focus on governance, safety, and professional accountability [7].

Quality assurance and standardisation are further strengthened by the work of van den Born-Bondt and colleagues, who address a critical and often underestimated aspect of aseptic preparation: the qualification of personnel involved in the visual inspection of parenteral medicinal products manufactured under GMP conditions. By proposing an adaptable test set and a feasibility-oriented approach, the study supports harmonised training strategies and contributes to improving the reliability of visual inspection as a safeguard against defects and particulate contamination [8].

In a complementary quality-driven contribution, Aquino and co-workers propose a practical analytical methodology for managing residual compounded antiblastic drugs, encompassing small molecules and monoclonal antibodies. Their work is particularly relevant for high-cost oncology preparations, reinforcing the importance of stability evidence to support safe handling and rational decision making in hospital pharmacy services [9].

A central theme of this Special Issue is the role of extemporaneous preparation in paediatric care, where the lack of age-appropriate medicines often necessitates formulation adaptation. Pai and Nahata provide a comprehensive and timely review of global necessities, challenges, and opportunities for paediatric extemporaneous formulations. Their review article critically discusses excipient safety, acceptability, beyond-use dating, and regulatory variability, reinforcing the view that paediatric compounding should be guided by evidence-based approaches rather than empirical adaptation alone [10].

Within paediatric neurology, Olivera and colleagues address a concrete therapeutic need by developing trihexyphenidyl orodispersible minitablets for the management of dystonia. This work illustrates how solid multiparticulate dosage forms can enable dose individualisation and improved acceptability, while offering robust performance and prolonged stability compared with less controlled liquid adaptations [11].

Beyond paediatrics, administration difficulties remain a major driver for extemporaneous formulation design, particularly in dysphagic patients. Logrippo and co-workers investigate gelled vehicles intended for oral drug administration in patients with swallowing impairment, demonstrating that the choice of carrier is a key determinant for swallowability, usability, and overall administration safety. This study reinforces that extemporaneous preparation often requires optimisation not only of the active ingredient but also of the administration vehicle [12].

A related patient-centred approach is presented by Trofimiuk and colleagues, who explore pharmacy-compounded oral gels as an alternative to conventional paediatric suspensions. By evaluating rheological and organoleptic properties alongside preservative-related considerations, the authors support the potential of semi-solid oral vehicles to improve acceptability and dosing reliability, particularly in home-care conditions where ease of administration and stability are essential [13].

Baratta and co-workers contribute a pragmatic example of “appropriate technology” compounding by developing a metronidazole suspension intended for paediatric use in developing countries. The study is framed within the A.P.P.A. project (Aid Progress Pharmacist Agreement), a collaborative initiative supporting the implementation of galenic laboratories and reproducible preparation protocols in contexts with limited healthcare resources. Using basic, low-cost excipients and a simple compounding approach, the work demonstrates how quality attributes such as content uniformity, dispersion behaviour, and pH stability can be systematically addressed even when advanced manufacturing infrastructure is unavailable. Beyond technical performance, the article effectively underscores the importance of palatability and acceptability in children, recalling the well-known message from the Walt Disney movie Mary Poppins that “a spoonful of sugar helps the medicine go down”, thereby linking formulation design to patient adherence and practical feasibility [14].

Dermatological compounding is addressed by Lacassia and colleagues through a comparative evaluation of five ready-to-use bases for topical propranolol hydrochloride preparations. This contribution highlights the practical relevance of ready-to-use vehicles as enabling tools for extemporaneous dermatological formulations, facilitating the incorporation of active substances while supporting acceptable texture, spreadability, and chemical-physical stability. The selected active ingredient further strengthens the clinical significance of the work, as topical propranolol represents a paradigmatic case of off-label use and drug repurposing in dermatology, where compounding may offer accessible and adaptable options when authorised alternatives are limited. By providing evidence-based criteria for vehicle selection, the study supports more standardised topical compounding practices and improved reproducibility across preparation processes [15].

An alternative oral platform is described by Jovičić-Bata and co-workers, who investigate liquid- and semisolid-filled hard gelatin capsules containing alpha-lipoic acid. This work illustrates how capsule-based compounding may provide flexible administration strategies while addressing handling and stability considerations, representing a practical dosage form option when conventional preparations are unsuitable [16].

Finally, the Special Issue expands the compounding perspective to nuclear medicine, where extemporaneous preparation is often intrinsic to clinical practice and must comply with stringent quality requirements. Rizzello and co-workers review methodologies for gallium-68 extemporaneous preparations and provide an overview of the relevant regulatory context. The article clearly illustrates how galenic practice supports the preparation of medicinal products not only for conventional therapy but also for advanced diagnostic and therapeutic procedures in nuclear medicine, where short radionuclide half-lives impose strict time windows and demand robust, standardised processes. By linking feasibility, quality control, and compliance, the contribution reinforces the essential role of compounding in enabling patient access to modern radiopharmaceuticals within routine hospital workflows [17].

## 3. Conclusions

This Special Issue offers a contemporary perspective on the role and relevance of extemporaneous formulation in modern healthcare. Extemporaneous formulation represents a pragmatic and clinically driven approach to personalised medicine, enabling the adaptation of therapies when authorised products do not meet individual patient needs. Its value relies on the rational selection of dosage forms and excipients, supported by stability and compatibility assessment, microbiological control, and fit-for-purpose quality assurance. When performed within appropriate regulatory and professional standards, compounding can provide safe and reproducible preparations across a broad range of applications. In this perspective, extemporaneous formulation is not merely a substitute for industrial medicines but a complementary pharmaceutical strategy that supports therapeutic continuity and optimises patient care.
